# The Role of PCR in Diagnosis of a Rare Appendicular Tuberculosis and Mini Literature Review

**DOI:** 10.1155/2016/8356708

**Published:** 2016-03-31

**Authors:** Asmerom Tesfamariam Sengal, Ahmed Abdalla Mohamedani, Hanan Hasaan Hussein, Alaa Kamal

**Affiliations:** ^1^Pathology Department, Faculty of Medicine, University of Gezira, P.O. Box 20, Wad Madani, Al Gezira, Sudan; ^2^Pathology Department, Gezira Medical Laboratory, Wad Madani, Al Gezira, Sudan

## Abstract

Tuberculosis is a prevalent public health problem especially in the poor developing countries and results in significant mortality. Albeit tuberculosis almost always affects any organ or system of the body, abdominal tuberculosis is less frequent; moreover, tuberculous appendicitis is very rare with an incidence estimated at about 0.1–0.6% of all gastrointestinal tuberculosis. The purpose of this report was to present an unusual case of primary tuberculous appendicitis and the approach used for accurate diagnosis as well as a current update on the disease. We are reporting a 30-year-old male who presented with acute abdominal pain, fever, and vomiting and was admitted with the clinical diagnosis of acute appendicitis. Patient was investigated thoroughly and histopathologic examination was strongly suggestive of tuberculous appendicitis; however, Ziehl-Neelsen (ZN) was negative in tissue section. To confirm the diagnosis, molecular biology [polymerase chain reaction (PCR)] study was performed from the formalin fixed paraffin embedded (FFPE) appendicular tissue and revealed presence of* Mycobacterium tuberculosis*. As there are numerous differential diagnoses in granulomatous lesions of appendix and due to the fact that appendicular tuberculosis is a rare phenomenon; verification etiologic agent is crucial for appropriate management of the disease.

## 1. Introduction

Tuberculosis (TB) is one of the most common causes of morbidity (281/100,000 people) and mortality (1.5 million annually) among infectious diseases in the world and with the advent of HIV/AIDS it is becoming a serious health problem. About two-thirds of the world population is presumed to be infected with* Mycobacterium*; however only 10% of those infected individuals develop the disease. The burden of the disease is significantly higher in African and Asian countries [1]. Tuberculosis virtually involves any organ of our body. Pulmonary tuberculosis is the most common and accounts for about 80% of clinical presentations. Extrapulmonary tuberculosis (EPTB) varies from region to region; however, the common sites in descending order are lymph node, genitourinary, bone and joint, miliary tuberculosis, meningeal tuberculosis, and abdominal tuberculosis [2].

Abdominal tuberculosis (ATB) is the 6th most common EPTB and accounts for about 1–3% of all tuberculosis in immunocompetent individuals. Its epidemiology varies depending on many factors such as age, sex, socioeconomic factors, immune status, and* Mycobacterium tuberculosis* genotype. Ileocecal tuberculosis is the most common accounting for about 75% of all cases of ATB [2, 3]. Though it is thought to be a relatively rare cause of morbidity and mortality and the prevalence is decreasing in many parts of the world due to sanitation improvement and routine milk pasteurization, this is not the case in some developing countries like Sudan with many agrarian society and poor sanitation still in place. However, appendicular tuberculosis and in particular an isolated appendicular TB is an extremely rare clinical phenomenon with incidence ranging between 0.1 and 0.6% of all gastrointestinal tuberculosis [4, 5]. We are presenting a case of acute abdomen diagnosed as tuberculous appendicitis. To the authors' knowledge, this was the first case to be reported in the region.

## 2. Case Report

The patient is a 30-year-old male, an agrarian from the rural area of the Gezira state, Sudan; he presented with right lower quadrant of abdominal pain, fever, and vomiting for 3 days. He was apparently healthy prior to his present illness and denied any contact history with known case of TB. The patient had no chronic illnesses and he is not alcoholic. Physical examination revealed rebound tenderness on the right lower abdomen, but no other pertinent findings on other systems.

**Figure 1:**
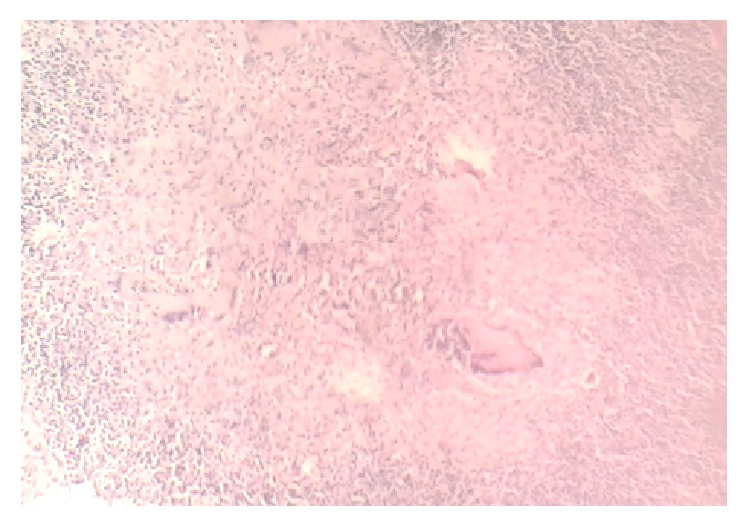
Microphotography ×100 H/E typical granuloma with Langhans multinucleated giant cells.

**Figure 2:**
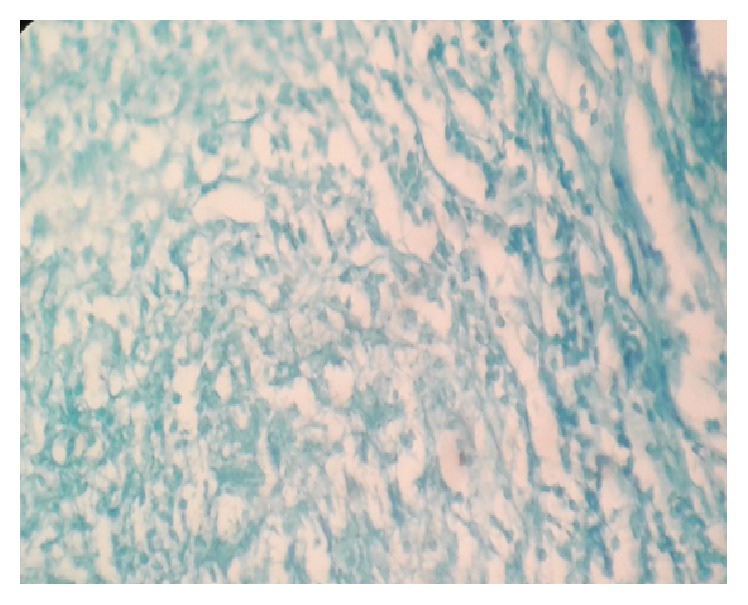
Microphotography ×400 ZN negative.

He was admitted to the emergency department with clinical diagnosis of acute appendicitis and investigated routinely for complete blood count (CBC), urine analysis, and stool examination for ova/parasites and all these laboratory studies were unremarkable. Emergency appendectomy was done and intraoperative findings showed that the appendix was congested. No other findings were found. The appendix was removed and sent for histopathologic examination. On gross examination, the appendix was 6 cm long, was mildly congested, and had smooth outer surface and serial sectioning showed narrow lumen filled with some necrotic material. Routine haematoxylin/eosin histologic examination showed a granulomatous lesion with mucosal ulceration involving the submucosa and muscular layers. It showed epithelioid cells, Langhans multinucleated giant cells, and mononuclear cells including lymphocytes in Figure 1. On histologic examination Ziehl-Neelsen (ZN) stain was negative for acid fast bacilli (Figure 2).

**Figure 3:**
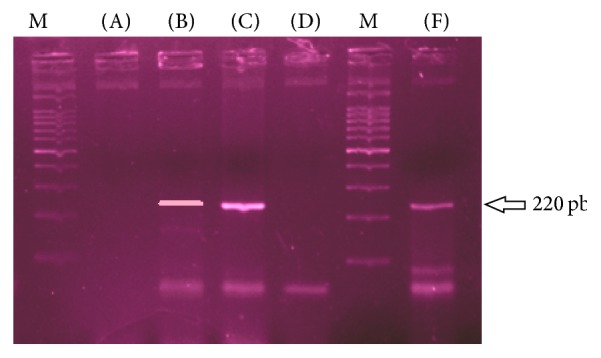
Result of amplified DNA product run on 1.5% agarose gel electrophoresis. M is the migration DNA ladder. Lane (A) is water as negative control, (B) is positive control, (C) is positive case from smear positive sputum, (D) is negative clinical cases, and (F) is our case from the appendicular tissue showing a positive 220 bp ladder.

To confirm diagnosis polymerase chain reaction (PCR) was performed following the standard instruction manual and protocol of iNtRON Biotechnology G-spin*™* total DNA extraction kit [6] from the formalin fixed paraffin embedded appendicular tissue. DNA amplification was done using thermocycler PCR (TC-3000X, Germany) with* Mycobacterium tuberculosis* multidetection kit (IS6110, IS1081, 16s) (iNtRON Biotechnology, Inc.), ready premix reagents following the protocol meticulously [7]. After 30-cycle amplification, the product with its positive Multi-TB and negative (distil water) control was run in 1.5% agarose gel electrophoresis and stained with nucleic acid stain and result was identified on ultraviolet transilluminator and PCR product was found positive for a 220 bp molecular ladder as noted in Figure 3, Lane F, DNA ladder.

Once the diagnosis was proved anti-TB first line treatment according to the direct observation treatment supervision (DOTS) strategy was initiated and currently he is on the third month of the second phase of treatment and has no obvious infirmity.

## 3. Discussion

There is paucity of data regarding appendicular TB as there are only few case reports published in the medical literature. In 155 case reports reviews the lowest and highest ages reported are 2 and 60 years, respectively, with a mean age of 27 and no sex preferences [8]. Tuberculous appendicitis could be primary or secondary with other concomitant infections in the body. The exact mechanism how the appendix gets infected is not clear; however, some authors suggest three possible ways: through the intestinal content contaminated with swallowed sputum or milk in case of* M. bovis*, direct extension from adjacent intestinal structures mainly caecum and rarely genitourinary, and by haematogenous or lymphatic spread [2, 3]. The uncommon occurrence of appendicular TB could be explained due to the lack of direct communication of appendix to the intestine. Our case is probably a primary appendicular TB as there was no evidence of any other focus of infection in the intestine or any other organ. As tuberculosis is a systemic disease it might not be necessary to screen retrospectively once a patient is diagnosed with tuberculosis appendicitis, unless clinicians are curious and wanted to rule out other secondary foci of infection, as the management does not change; however, it is worth screening for HIV.

Clinical diagnosis of tuberculous appendicitis is puzzling as the symptoms and signs are nonspecific. There are three types of clinical categories which have been documented. The first is acute presentation that mimic pyogenic/phlegmonous appendicitis presenting with fever, vomiting, and right iliac fossa pain. The second is a chronic course with vague abdominal pain with or without bowel habit change and appendicular mass, and the third is asymptomatic covert and incidental finding in appendectomy specimen done for other purposes [4, 5]. In our case the presentation was consistent with the first category. In patients who present with acute abdomen it is not clearly understood why the onset is flared up acutely. This needs more detailed systemic investigation; however, it is probably as a result of the ongoing acute-on-chronic inflammatory process and some exaggerated immune reaction produces inflammatory mediators and cytokines that are responsible for the development of the symptoms.

The reliable method for evaluation of appendicular TB is histological examination with routine H/E in light microscope to reveal the caseous tubercle granuloma. The fact that there could be some mimicking granulomatous lesions such as Crohn's disease, sarcoidosis, and parasitic or mycosis granuloma should be borne in mind. ZN stain is a further step to confirm the tubercle bacilli in the tissue in clinical practice. Despite the routine use of ZN stain in histologic examination it is less sensitive and could give a false negative result as in the case of our patient which was negative for ZN but was confirmed by PCR to be positive. The plausible explanation for this could be that the mycolic acid rich in fat on the cell wall of* Mycobacterium* (which react with the primary stain, carbol fuchsin) might be damaged in process of fixation and processing by formalin and xylene and hardly stains with ZN in FFPE tissue as supported by a study finding from Japan [9]. Even though PCR is expensive for routine clinical diagnosis, it is relatively rapid, highly sensitive, and specific method of assessment of tuberculosis in FFPE tissue samples [10].

Finally, the authors recommend careful histologic examination of every appendectomy specimen in order not to miss the rare cases and delay treatment of appendicular tuberculosis especially in areas with high burden of TB.
